# The Facile Deposition of Pt Nanoparticles on Reduced Graphite Oxide in Tunable Aryl Alkyl Ionic Liquids for ORR Catalysts

**DOI:** 10.3390/molecules27031018

**Published:** 2022-02-02

**Authors:** Dennis Woitassek, Swantje Lerch, Wulv Jiang, Meital Shviro, Stefan Roitsch, Thomas Strassner, Christoph Janiak

**Affiliations:** 1 Institut für Anorganische Chemie und Strukturchemie, Heinrich-Heine-Universität Düsseldorf, 40225 Düsseldorf, Germany; woitasse@hhu.de; 2 Physikalische Organische Chemie, Technische Universität Dresden, 01062 Dresden, Germany; swantje.lerch@tu-dresden.de; 3 Forschungszentrum Jülich GmbH, Institute of Energy and Climate Research, IEK-14: Electrochemical Process Engineering, 52425 Jülich, Germany; j.wulv@fz-juelich.de; 4 Institut für Physikalische Chemie, Universität zu Köln, 50939 Köln, Germany; sroitsch@uni-koeln.de

**Keywords:** platinum, nanoparticles, electrochemistry, oxygen reduction reaction, reduced graphite oxide, microwave, ionic liquid, tunable aryl alkyl ionic liquid

## Abstract

In this study, we present the facile formation of platinum nanoparticles (Pt-NPs) on reduced graphite oxide (rGO) (Pt-NP@rGO) by microwave-induced heating of the organometallic precursor ((MeCp)PtMe_3_ in different tunable aryl alkyl ionic liquids (TAAIL). In the absence of rGO, transmission electron microscopy (TEM) reveals the formation of dense aggregates of Pt-NPs, with primary particle sizes of 2 to 6 nm. In contrast, in the Pt-NP@rGO samples, Pt-NPs are homogeneously distributed on the rGO, without any aggregation. Pt-NP@rGO samples are used as electrode materials for oxygen reduction reaction (ORR), which was assessed by cyclic voltammetry (CV) and linear sweep voltammetry (LSV). The electrochemical surface area (ECSA) and mass-specific activity (MA) increase up to twofold, compared with standard Pt/C 60%, making Pt-NP@rGO a competitive material for ORR.

## 1. Introduction

Platinum nanoparticles (Pt-NPs) are important catalysts and are often used as benchmark materials in the field of electrochemistry for the oxygen reduction reaction (ORR) [1,2,3,4,5], the hydrogen evolution reaction (HER) [6,7], and the methanol oxidation reaction (MOR) [8,9,10]. In general, the stability, catalytic activity, and chemical selectivity of Pt-NPs depend strongly on their size, shape, alloy composition, surface structure, and surface accessibility [2,5,11]. It is, therefore, important to control these parameters via the chosen synthetic method and, if possible, influence multiple parameters at the same time during NP synthesis [2,5,11].

Smaller Pt-NP sizes show higher metal-mass-based catalytic activity than larger particles or bulk-material [5,11,12]. At the same time, a crucial issue for NPs is their tendency to coalescence, which can occur as agglomeration or Ostwald ripening, increasing their size and reducing the catalytic activity, thus requiring stabilizers [13,14,15].

We have recently reported the wet-chemical synthesis of Ru- and Ir-NPs in tunable aryl alkyl ionic liquids (TAAILs) based on the 1-aryl-3-alkyl-substituted imidazolium motif [16]. Ionic liquids (ILs) are salts with a melting point below 100 °C and can be used as solvents and as stabilizing agents for M-NPs, due to their high ionic charge, polarity, dielectric constant, and electrostatic and steric interaction with the M-NPs [13,14,15,17,18]. All used TAAILs were suitable for the formation of very small (<5 nm) NPs, which were stable over months in the IL medium. An interesting observation was that the nanoparticle separation and aggregation varied strongly as a function of the aryl substituent of the TAAILs.

In addition, Supporting Materials can stabilize M-NPs, allowing for easier handling of the M-NPs and providing for additional tuning options for applications. Carbon materials as support for Pt-NPs are commonly used in the field of electrochemistry because of their high electrical conductivity, low weight, low cost, easy production, and manifold structures [6,19]. Our group has already shown the usage of (thermally) reduced graphite oxide (rGO, also known as TRGO) as M-NP support [20,21], which also offers remarkable properties as support for electrode materials [22,23,24]. rGO increases the electrochemical activity of Pt-NPs [19], stabilizes very small M-NPs [25,26], and offers improved protection for supported NPs against poisoning, compared with available standard carbon black materials [20]. Since rGO provides less functional groups that can interact with M-NPs than graphite oxide, the deposition of Pt-NPs on rGO (Pt-NP@rGO) in ILs remains difficult and was only successful after additional rGO functionalization with thiol groups [20] to improve the interaction between Pt-NPs and its support. Alternatively, porous organic polymers with donor atoms such as covalent triazine frameworks (CTFs) were successfully used as support [27].

In this article, we present a simple and facile way to deposit Pt-NPs in situ on rGO during a microwave-assisted reaction in ionic liquids. Novel imidazolium-ILs, which are distinguished aryl and alkyl substitutions, are used as reaction media as well as stabilizing agents. We also show that the obtained Pt-NP@rGO are electrochemically active as heterogeneous catalysts for ORR, a vital reaction for proton exchange membrane fuel cells (PEMFC).

## 2. Results

### 2.1. rGO and TAAIL Presentation

rGO was synthesized in a two-step oxidation–thermal reduction process according to procedures by Hummers [28], as described in more detail in our previous studies [20,21], with the thermal reduction occurring at 400 °C. CHNS elemental analysis showed a weight percentage (wt%) of 80% carbon, which indicates that functional oxygen-containing groups still remain on the surface of rGO [21]. The exact values can be found in the Supporting Information (SI), Table S2.

**Scheme 1:**
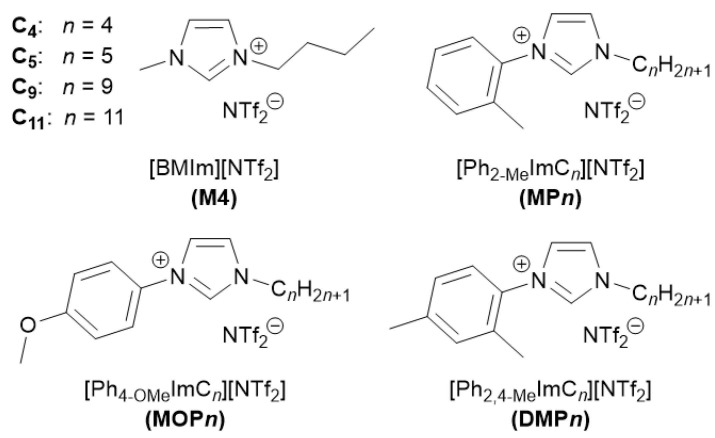
Structures of utilized tunable aryl alkyl ionic liquids (TAAIL) with 1,3 disubstituted imidazolium cations and the bis(trifluoromethylsulfonyl)imide anion. The abbreviation is derived from the phenyl substitution and the alkyl chain length.

The synthesis and characterization of the TAAILs used here were described elsewhere [15,29,30]. TAAILs refer to 1-n-alkyl-3-arylimidazolium ILs (Scheme 1). The length of the n-alkyl chain varies here between 4 and 11 carbon atoms, and the aryl groups were 4-methoxy-, 2,4-dimethyl, 4-bromo- and 2-methylphenyl.

The TAAILs can be obtained on a large gram scale, and the synthesis allows manifold variations of the aryl and alkyl groups [15,29,30]. The anion purity of the TAAILs was characterized via ion chromatography and has always been found above at least 97% and more often 99%. Thermogravimetric analysis (TGA) of the TAAILs showed decomposition between 400 °C and 426 °C, which allows them to be used in the Pt-NP-forming microwave reactions at ~200 °C.

### 2.2. Pt-NP Synthesis and Characterization

Following previous research [13], (η^5^-methylcyclopentadienyl)trimethylplatinum(IV) ((MeCp)PtMe_3_) was used as a Pt precursor, which can be decomposed in various ways (thermal, photolytic, sonolytic) at relatively mild conditions and without any additives [13,20]. The decomposition of the precursor produces volatile side products, which do not contaminate the NP surface [31,32,33].

**Scheme 2:**
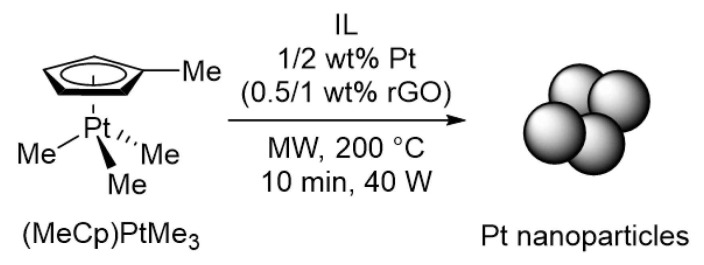
Reaction conditions for the synthesis of platinum nanoparticles (Pt-NPs) via microwave-induced heating in ionic liquids (ILs). The mass of the Pt precursor was set to achieve 1 wt% or 2 wt% Pt-NP in the IL dispersion upon complete decomposition (0.5 wt% or 1 wt% reduced graphite oxide (rGO) used for Pt-NPs stabilized on rGO (Pt-NP@rGO) in brackets).

The general synthetic procedure of the Pt-NPs is depicted in Scheme 2. Fixed amounts of Pt precursor and rGO were suspended in the IL to achieve 1 wt% or 2 wt% Pt-NP and 0.5 wt% or 1 wt% rGO in the IL dispersion. After stirring overnight and for 3 h of sonication, the dispersion was placed in a microwave reactor, heated at 40 W to 200 °C, and kept at this temperature for 10 min. Black Pt-NP@rGO dispersions were reproducibly obtained.

**Figure 1:**
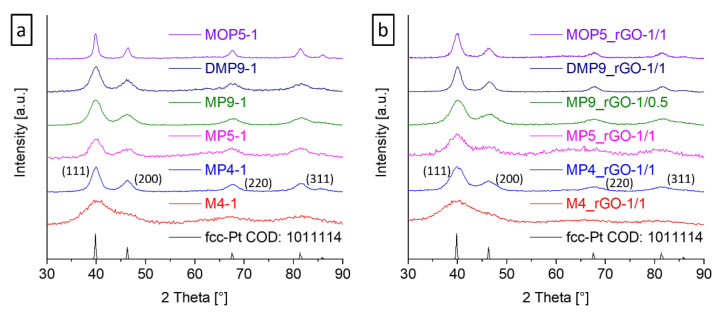
Powder X-ray diffraction (PXRD) patterns of synthesized Pt-NPs (**a**) and Pt-NP@rGO (**b**) in ILs, together with the simulation for fcc-Pt and its indexed reflections (Crystallographic open database fcc-Pt: 1011114). The -X and -X/Y numbers refer to wt% Pt (X) and wt% rGO (Y) in samples. Additional PXRD patterns can be found in Figures S2–S6 in the Supporting Information (SI).

Selected samples and their respective particle size determined via transmission electron microscopy (TEM) are shown in Table 1. In the Supporting Information, Table S1, a full list of prepared samples is given. The nanocrystallinity of the Pt particles was verified by powder X-ray diffraction (PXRD) (Figure 1 and Figures S2–S6 in the Supplementary Information). The Pt-NPs form in the face-centered cubic (fcc) structure, typical for crystalline Pt. The comparison of the crystallite sizes of the NPs calculated with the Scherrer equation (Section 4.1, Equation (1)) and the Pt-NP sizes determined via TEM showed a very good agreement (Table S1, Supplementary Information). Furthermore, thermogravimetric analysis (TGA) of elected samples (Figure S28 in the Supplementary Information) gives a residual Pt mass between 41 wt% and 54 wt% or a reciprocal combined rGO and IL content between 46 wt% and 59 wt%. The mass-temperature profile in TGA did not allow a distinction between rGO and IL. From an estimate of the IL content by CHNS analysis (Table S2), the rGO mass was then approximated between 40 wt% and 54 wt% (Table S4).

Similar to Ir-NPs, Ru-NPs [15], and Pt-NPs [13], the microwave-assisted heating of the platinum metal precursor (MeCp)PtMe_3_ in ionic liquid dispersion results in small M-NP sizes, largely between 2 nm and 5 nm. The efficient energy uptake of ILs enables fast heating and results in rapid decomposition of the Pt precursor with a high nucleation rate of Pt-NPs, which themselves then absorb microwave radiation. This leads to “hot spots”, with a further increase in localized temperature. Small particle sizes can only be achieved if the TAAIL is able to stabilize the newly formed NPs from the very beginning of the growth process. The Pt-NPs show similar sizes mostly independent of the choice of TAAIL and the wt% Pt to IL (Table S1, Supplementary Information). An exception is the TAAIL with the 4-methoxyphenyl substituent (MOP5 to MOP11), where also larger particles, up to 6 nm, were obtained.

### 2.3. Pt-NPs Aggregation in TAAILs

As seen before for Ru- and Ir-NPs, [15], the TAAILs allow the microwave-induced synthesis of Pt-NPs, but strong Pt-NP aggregation was observed (in the absence of rGO) for all TAAILs, independent of the alkyl chain length or the aryl substitution (Supplementary Information, Figures S7–S27), whereas the comparative IL [BMIm][NTf_2_] gave well-separated NPs (Figure 2 top, Figure S7). Different from the synthesis of Ru- and Ir-NPs, [15] even the 4-methoxyphenyl- and 2,4-dimethylphenyl-TAAIL did not prevent strong aggregation.

**Figure 2:**
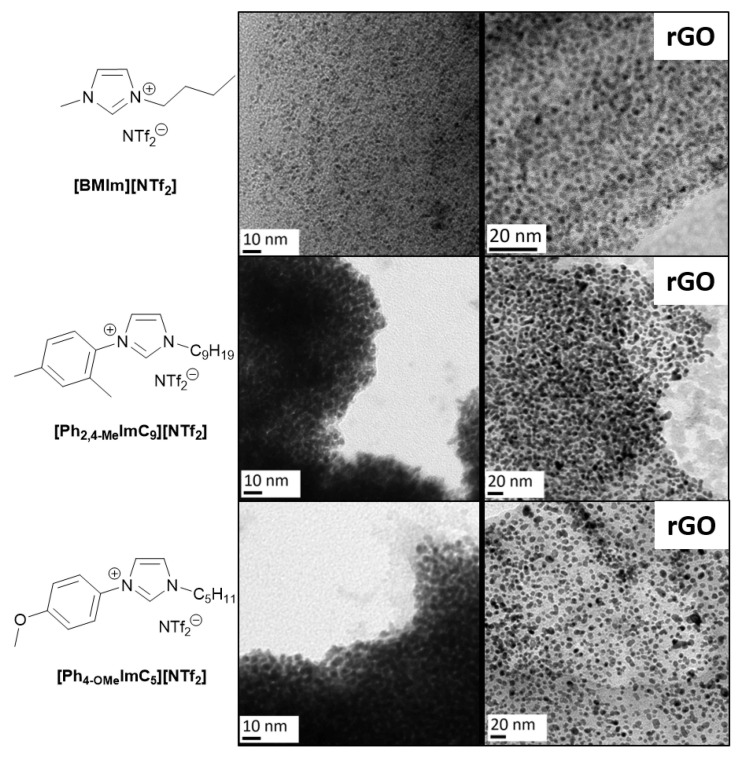
TEM images of Pt-NPs and Pt-NP@rGO composites obtained in the IL (from top to bottom) [BMIm][NTf_2_] (**M4-1, M4_rGO-1/1**) and the TAAILs [Ph_2,4-Me_ImC_9_][NTf_2_] (**DMP9-1, DMP9_rGO-1/1**) and [Ph_4-OMe_ImC_5_][NTf_2_] (**MOP5-1, MOP5_rGO-1/1**). TEM images for Pt-NPs are shown on the left and for Pt-NP@rGO composites on the right. The rGO sheets on which the Pt-NP are deposited are clearly seen by their wrinkles at lower magnification (e.g., Figures S10, S12, S16, S17, S19, S25, and S27 in the Supplementary Information).

**Figure 3:**
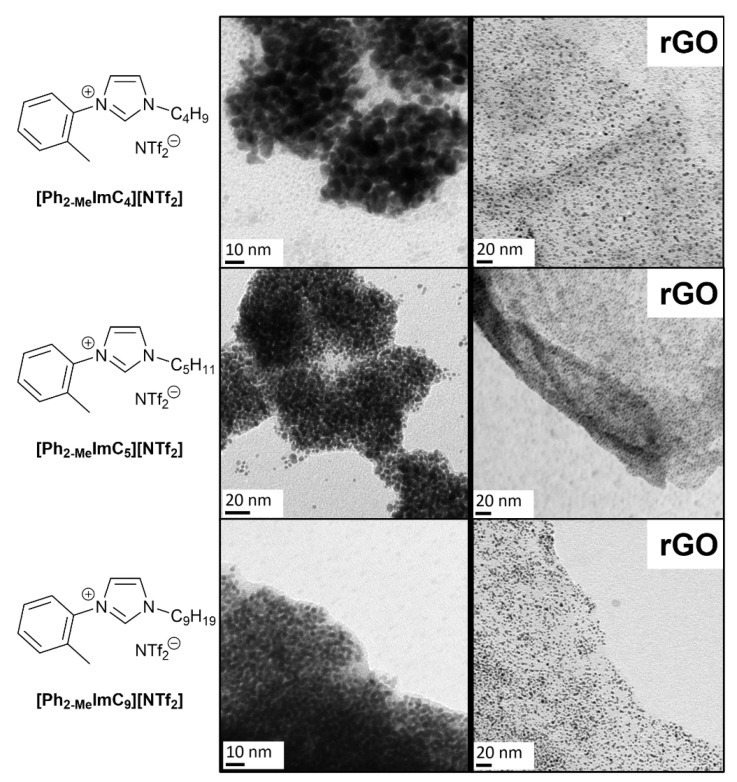
TEM images of Pt-NPs and Pt-NP@rGO composites obtained in the TAAILs (from top to bottom): [Ph_2-Me_ImC_4_][NTf_2_] (**MP4-1, MP4_rGO-1/1**); [Ph_2-Me_ImC_5_][NTf_2_] (**MP5-1, MP5_rGO-2/1**); [Ph_2-Me_ImC_9_][NTf_2_] (**MP9-1, MP9_rGO-1/0.5**). TEM images for Pt-NPs are shown on the left and for Pt-NP@rGO composites on the right. The rGO sheets on which the Pt-NP are deposited are clearly seen by their wrinkles at lower magnification (e.g., Figures S10, S12, S16, S17, S19, S25, and S27 in the Supplementary Information).

Aggregation, however, could be prevented upon deposition on rGO by performing the (MeCp)PtMe_3_ precursor decomposition in the presence of rGO. Representative TEM images of Pt-NPs synthesized in different TAAILs without and with rGO are shown in Figure 2 and Figure 3. The rGO-free samples consist of highly aggregated Pt-NPs, with large, dense structures, with dimensions of several tens of nanometers. The degree of aggregation for the Pt-NPs appears independent of the TAAIL.

In previous research, we discussed the necessity of thiol functional groups on rGO to deposit Pt nanoparticles as Pt-NP@rGO when using the IL [BMIm][BF_4_] [20]. In contrast, with the TAAILs used in this study (and also with the [NTf_2_]-IL M4 (Figure S8 in the Supplementary Information)), Pt-NPs can be effectively stabilized on rGO without thiol functional groups, resulting in a rather homogenous layer of Pt-NPs on the surface of the rGO (Figure 2 and Figure 3). Compared with the rGO-free samples, nearly no additional agglomeration of NPs can be found, which indicates that the NPs are effectively anchored to prevent aggregation.

The stability of prepared Pt-NPs and Pt-NP@rGO samples was followed by storing them dried under air at room temperature for at least 6 months. PXRD patterns and the calculated crystallite sizes (Equation (1)) from representative samples are given in Table S3 and Figures S3–S6 in the Supplementary Information. Additionally, a TEM image of an rGO-free and a Pt-NP@rGO sample after 1 year did not show significant particle growth or changes in aggregation (Figures S26 and S27 in Supplementary Information).

### 2.4. Electrochemical Catalysis

**Figure 4:**
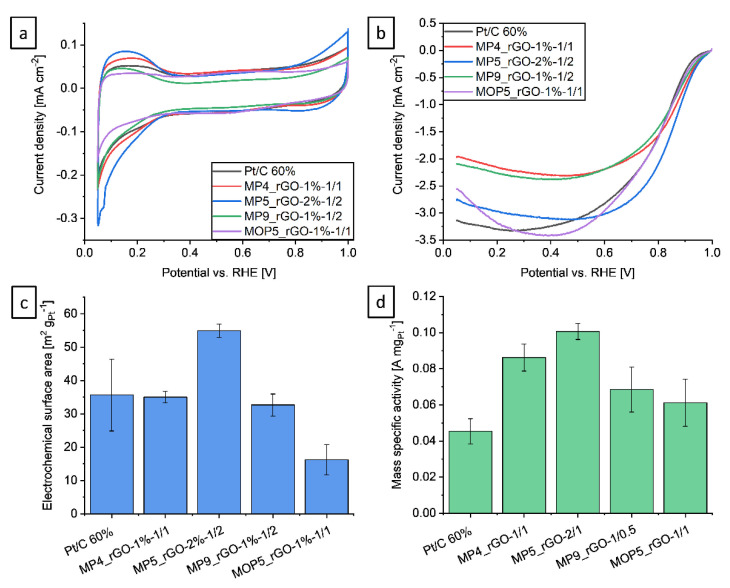
Results obtained via electrochemical measurements: (**a**) averaged cyclic voltammetry (CV) in N_2_-atmosphere and at 20 mV s^−1^ sweep rate of the four samples **MP4_rGO-1/1**, **MP5_rGO-2/1**, **MP9_rGO-1/0.5**, **MOP5_rGO-1/1**, and a Pt/C 60% standard material; (**b**) averaged oxygen reduction reaction (ORR) polarization curves (linear sweep voltammetry (LSV)) of the same samples in O_2_-saturated atmosphere at 10 mV s^−1^ sweep rate. From these data, (**c**) the electrochemical surface area (ECSA) and (**d**) the mass-specific activity (MA) at 0.9 V were calculated.

The electrochemical performance of obtained Pt-NP@rGO for the ORR was examined with commercial Pt/C 60% as internal benchmark material in comparison, to reduce deviations by the use of an external standard. As samples, **MP4_rGO-1/1**, **MP5_rGO-2/1**, **MP9_rGO-1/0.5**, and **MOP5_rGO-1/1** were chosen because of their low size and homogeneity, as depicted in Figure 4. The cyclic voltammogram (CV) curves were recorded in N_2_-saturated 0.1 M HClO_4_, with a sweep rate of 20 mV s^−1^ and, after 50 activation cycles, against reversible hydrogen electrode (RHE, Figure 4a). Afterward, ORR polarization curves (linear sweep voltammetry (LSV)) with a sweep rate of 10 mV s^−1^ were recorded in O_2_-saturated 0.1 M HClO_4_. These measurements were repeated three times each, and the respective combined average CVs and LSVs are shown in Figure 4a,b. The electrochemical surface area (ECSA) was determined by integrating the hydrogen underpotential deposition (H_upd_) charge (H_upd_ charge to surface area conversion constant: 210 μC cm^−2^) in the obtained CVs, and, with the obtained LSVs, mass-specific activities (MAs) at 0.9 V were calculated. The obtained ECSA and MA are compared in Table 2 to the results of Pt-NPs deposited on rGO via different reaction methods from previous studies of Badam et al. [34], Daş et al. [35], and Teran-Salgadon et al. [36].

All CVs of the materials, shown in Figure 4a, have well-pronounced underpotential hydrogen deposition–desorption areas between 0.05 V and 0.40 V, with **MP4_rGO-1/1** and **MP5_rGO-2/1** having a larger area than the commercial Pt/C catalyst. No contaminations or side reactions are recognizable in the CVs. The calculated ECSA, shown in Figure 4c, reveals the highest value for **MP5_rGO-2/1**, with a 1.5-fold increase, compared with Pt/C 60%, while the other samples show similar values as the benchmark material. This shows that rGO can be effectively used as in situ carbon source, in addition to the most commonly used Vulcan XC-72R, which is only added for preparing the catalyst ink. The ORR polarization curves (Figure 4b) of the samples show better onset potentials than the benchmark material and different current density plateaus between 0.20 V and 0.60 V. The presented materials reach their respective plateau at higher voltages but at lower currents than Pt/C 60%. From the LSVs the mass-specific activity (MA) is calculated (Figure 4d). The highest MA is achieved by **MP5_rGO-2/1**, with a promising twofold increase, compared with the Pt/C 60% benchmark, while **MP4_rGO-1/1**, **MP9_rGO-1/0.5**, and **MOP5_rGO-1/1** also show an increase of more than 1.8-fold, 1.4-fold, and 1.25-fold, respectively, demonstrating a better performance of Pt-NP@rGO for ORR than Pt/C 60%. Calculated Tafel plots of the samples, shown in Figure S29 in the Supplementary Information, are similar to the Tafel plot of Pt/C 60% and indicate a four-electron pathway, with the adsorption of oxygen as a rate-limiting step [37]. The Koutecký–Levich plot calculated from LSVs of **MP4_rGO-1/1** at different rotation speeds (Figures S30 and S31 in the Supplementary Information) also shows a dominant four-electron pathway toward the ORR process.

A comparison of previous research on Pt-NP@rGO shows the highest ECSA and MA for Pt-NP decorated on tetradecyl-methyl-imidazolium ionic liquid-treated graphene (Pt-TMIm-rGO) by Badam et al. [34], albeit with a similar ECSA to the 55 m^2^ g_Pt_^−1^ of **MP5_rGO-2/1**. Notably, the **MP_rGO** samples have higher ECSA values, compared with the results of the Pt/rGO probes of Daş et al. [35] and Teran-Salgadon et al. [36], much higher MA values than obtained by Daş et al. [35] and similar to slightly smaller MA values than given by Teran-Salgadon et al. [36]. From the comparison, it becomes evident that ILs can significantly increase the catalytic activity of Pt-NP@rGO samples.

### 2.5. Stability Testing

**Figure 5:**
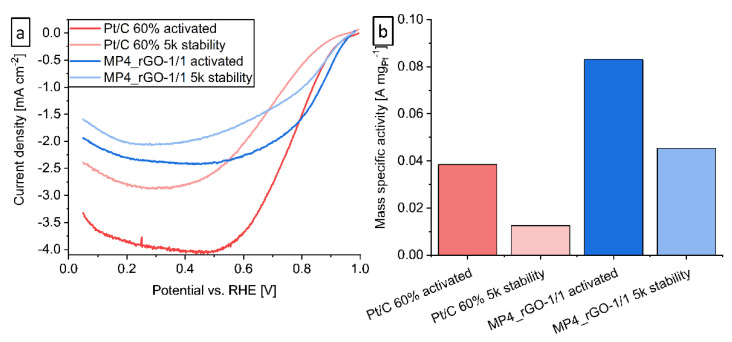
Electrochemical activity of the sample **MP4_rGO-1/1** and Pt/C 60% as reference: (**a**) obtained linear sweep voltammetry at 10 mV s^−1^ sweep rate after the activation and after 5k cycles; (**b**) calculated mass-specific activity at 0.9 V after activation and after 5k cycles.

**MP4_rGO-1/1**, the most active sample for ORR, was further analyzed with an electrochemical stability test. After the material was activated via CV (20 cycles), 5k cycles between 0.5 and 1.0 V_RHE_ were carried out. The obtained LSVs and calculated MA after activation and after 5k cycles are shown in Figure 5 and in Table 3.

**Figure 6:**
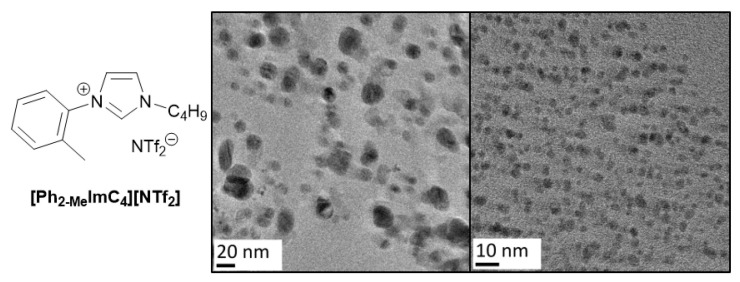
TEM images of the sample **MP4_rGO-1/1** obtained after 5k stability test. Images were measured with an FEI Titan 80–300 TEM.

The LSV of **MP4_rGO-1/1** after 5k cycles shows a decrease in MA, by roughly 45%, while Pt/C 60% loses more than 60% of MA. The activity loss of the sample can be explained with the aid of TEM images obtained after the stability test, shown in Figure 6. When compared with the freshly prepared NPs (Figure 3), the Pt-NP sample lost its small-size homogeneity, with also larger NPs, for which sizes of over 20 nm were clearly visible. Since this increase in size was not observed via PXRD and TEM for dried Pt-NPs, which were stored in air at room temperature for half a year and up to one year, we assume that this NP growth is linked to the ORR.

We conclude that the NPs can be detached from the stabilizing rGO support during potential cycling and start to agglomerate, losing their electrochemical activity. Since small Pt-NPs remain on the GC surface and show electrochemical activity, the exact position of the NP on the rGO might have an influence on this process, with NPs attached to inner layers of the rGO being more stable. Although not visible, in images with small Pt-NPs, the resolution to depict the rGO can be difficult because stacked or strongly wrinkled rGO sheets might have been removed during ink preparation or electrocatalytic reactions. A complete detachment of Pt-NPs of the rGO would be unlikely because the activity loss would be much stronger with more NP agglomerates and no small NPs detectable.

## 3. Conclusions

Small Pt-nanoparticles (Pt-NPs) as dispersed in ionic liquids and in situ deposited on reduced graphite oxide (rGO) (Pt-NP@rGO) can be obtained during a fast and effective microwave-assisted thermal decomposition of (MeCp)PtMe_3_ without and with rGO added and tunable aryl alkyl ionic liquids (TAAILs), as solvent and stabilizer. The particle size of the obtained Pt-NPs can vary slightly, depending on the TAAIL, and lies between 2 ± 1 nm and 6 ± 2 nm. Independent of the particle size, the Pt-NPs build dense aggregates. When comparing the TAAILs as reaction media with each other, the length of the alkyl chain or the aryl group only has an insignificant effect on the obtained particle size and aggregation.

For samples containing rGO, TEM images show that Pt-NPs build a layer on top of the rGO surface and maintain a homogenous size. In addition, no particle aggregation is observable, which demonstrates that rGO effectively anchors the obtained NPs and prevents their agglomeration. Pt-NPs and Pt-NP@rGO samples show good stability over at least 12 months, with no increase in particle size and no further agglomeration observable.

Pt-NP@rGO samples on glassy carbon electrodes for the oxygen reduction reaction (ORR) exhibit an up to a twofold increase in electrochemical surface area (ECSA) and mass-specific activity (MA), compared with Pt/C 60%. **MP5_rGO-2/1** achieved the best performance of the analyzed Pt-NP@rGO samples, which all appear promising for ORR and are worthy of further investigation. Since Pt-NP sizes between 2 nm and 3 nm are known to be the most active size range for ORR, and TAAILs make this size range readily available, their use as reaction media for Pt-NP preparation asks for further investigations. An organometallic Pt precursor and TAAILs in the presence of carbon support, in combination with microwave-induced heating, offer fast access to Pt-based electrocatalysts on the nanoscale.

## 4. Materials and Methods

### 4.1. Chemicals and Equipment

The following chemicals were received from commercial sources: Pt/C (60 wt% Pt on Vulcan XC-72R, Sigma-Aldrich), Nafion 1100 W (Sigma-Aldrich), n-butyllithium (1.6 mol L^−1^ in hexane, Acros organics), methyllithium (1.6 mol L^−1^ in diethyl ether, Sigma-Aldrich), 1,2-dibromoethane (>98%, Fluka), potassium hexachloridoplatinate (IV) (97%, BLDpharm), potassium iodide (USP, BP, Ph. Eur. pure, pharma grade, PanReac Applichem), methylcyclopentadienyl dimer (95%, Acros Organics), and perchloric acid (70%, ACS reagent, Sigma-Aldrich). All chemicals were used as received without further purification.

Thermally reduced graphite oxide (rGO) was prepared in a two-step oxidation–thermal reduction process using natural graphite (type KFL 99.5 from AMG Mining AG, former Kropfmühl AG, Passau, Germany). The graphite oxidation procedure of Hummers and Offeman [28] was followed. rGO was obtained at a reduction temperature of 400 °C from graphite oxide. For further information, also see our previous studies [21,25].

Tuneable aryl alkyl ionic liquids (TAAILs) were obtained in a two-step synthesis at the group of Prof. Thomas Strassner, Technische Universität, Dresden. The first step was the alkylation of the aryl imidazoles by bromoalkanes to build the IL cations. As the second step, the anion (bromide) was exchanged with LiNTf_2_. For detailed information, see Supporting Information in Refs. [15,30].

(η^5^-methylcyclopentadienyl)trimethylplatinum(IV) ((MeCp)PtMe_3_) was synthesized and characterized after a method described by Xue et al. [13,33].

Transmission electron microscopy (TEM) measurements were performed with a JEOL-2100 Plus, a Zeiss LEO912, and an FEI Titan 80–300 TEM at 200 kV, 120 kV, and 300 kV accelerating voltage, respectively. The samples were prepared using 200 μm carbon-coated copper grids. Briefly, 0.05 mL of the NP/IL dispersion was dissolved in a 0.5 mL acetonitrile (ACN), and one drop of the diluted dispersion was placed on the grid. After 30 min, the grid was washed with 3 mL ACN and air dried. The images were analyzed by Gatan Microscopy Suite version 3.3, and the particle size distribution was determined from at least 200 individual particles at different positions on the TEM grid, with the same magnifications.

Powder X-ray diffractograms (PXRDs) were measured at ambient temperature on a Bruker D2-Phaser using a flat sample holder and Cu-Kα radiation (λ = 1.54182 Å, 35 kV). Diffrac.Eva V4.2 was used to evaluate PXRD data. Particle sizes were calculated with the Scherrer equation (Equation (1)) as follows:

*L* = *K* × λ/(Δ(2*θ*) × cos *θ*_0_) (1)

where *L* is the average crystallite size, *K* is the dimensionless shape factor, λ is the wavelength, Δ(2*θ*) is the full width at half maximum (FWHM) in radians, and *θ* is the Bragg angle.

A CEM-Discover SP microwave setup, with a power range of 0–300 W (±30 W) was used for all microwave reactions. Thermogravimetric analysis (TGA) was conducted with a Netzsch TG 209 F3 Tarsus device, equipped with an Al crucible applying a heating rate of 5 °C/min under a synthetic air atmosphere.

### 4.2. Synthesis of Pt-NPs in IL

As a general approach to obtain Pt-NPs, (MeCp)PtMe_3_ was put in a 10 mL microwave vessel and dispersed in the corresponding IL. The necessary amount of Pt precursor compared with IL was determined by the wt% of obtained Pt-NPs in IL with 100% conversion, which was set between 1 wt% and 2 wt%. The general batch size was further customized with roughly 300 mg, 500 mg, and 1.00 g IL. The dispersion was stirred for at least 6 h and then heated in the microwave (200 °C, 40 W, 10 min holding time). Afterward, 3 mL acetonitrile was added to the black dispersion, mixed, and centrifuged. The clear liquid phase was removed. This washing process was repeated until a colorless, clean solution could be removed (mostly four times). The residue was dried in a high vacuum, to obtain pure Pt-NPs. A list of all obtained Pt-NPs can be found in Table S1 in the Supplementary Information.

For the synthesis of Pt-NPs in IL with rGO, 0.5 wt% to 1 wt% rGO was additionally added into the microwave vessel, stirred overnight, and sonicated for 3 h. The following steps were carried out as mentioned before.

### 4.3. Electrochemical Measurements

For all measurements, a similar setup and procedure as in Beermann et al. were used [38]. A conventional three-electrode cell, with a Pt gauze as a counter electrode (Pt furled Pt 5 × 5 cm^2^ mesh), a reference electrode (reversible hydrogen electrode = RHE), and a glassy carbon-working electrode (5 mm diameter), was used. The working electrode was always lowered into the electrolyte under potential control at 0.05 V_RHE_. For all measurements, a 0.1 M HClO_4_ electrolyte solution, diluted from 70% concentrated HClO_4_ with Milli-Q water, was used. For monitoring, BioLogics potentiostats SP-150 or SP-200 were used.

Fresh inks were prepared from the NPs as the electrochemically active material. For the Pt-NP@rGO inks, NPs containing 1 mg Pt were mixed with 0.5 mL isopropanol, 2.5 mL water, and 10 µL nafion 5% and sonicated for at least 30 min. Since the samples already contained rGO as carbon support, the catalyst ink did not contain additional carbon. Next, 10 µL of the ink was deposited onto the working electrode, to achieve a loading of 10 µg_Pt_ cm^−2^, and dried for 10 min.

After the electrolyte was purged with N_2_ for 20 min, the electrochemical activation was performed via potential cycling between 0.050 and 0.925 V_RHE_, with a scan rate of 100 mV s^−1^ for 50 cycles under a N_2_-protected atmosphere. The H-adsorption-based electrochemically active surface area (ECSA) was determined, with the last cycle recorded as 20 mV s^−1^, before the activity measurements were selected. The cyclic voltammetry (CV) was carried out by cycling between 0.05 and 1.0 V_RHE_, with a scan rate of 20 mV s^−1^, under a N_2_ atmosphere. The charge values (Q_H_) were calculated by integrating the respective CV between 0.05 V and 0.4 V. The measured Q_H_ values were normalized with respect to the theoretical value of Q_H_^theo^ = 210 μC cm^−2^, which is assuming a one-electron transfer between one H atom and one Pt atom. To determine the catalytic activity of the catalysts, linear sweep voltammetry (LSV) was measured in an oxygen saturated electrolyte (after 15 min of bubbling) in a potential range between 0.05 and 1.0 V_RHE_, with a scan rate of 10 mV s^−1^ and a rotation speed of 1600 rpm. These measurements were repeated three times. The kinetic currents were calculated using (Equation (2)) as follows:

*I*_k_ = (*I*_lim_ × *I*)/(*I*_lim_ − *I*) (2)

where *I* is the measured current density, *I*_lim_ is determined in the diffusion-limited current area, and *I*_k_ is the calculated kinetic current density. All presented current densities are iR corrected, where the uncompensated ohmic resistance (R) was determined by potential electrochemical impedance spectroscopy (PEIS), at 0.4 V_RHE_. Additional rotation speeds of 225, 400, 625, 900, and 1225 rpm were measured for the sample **MP4_rGO-1/1**.

Stability measurements were performed via potential cycling between 0.5 and 1.0 V_RHE_, with a scan rate of 50 mV s^−1^, in N_2_-saturated 0.1 M HClO_4_, with 0 rpm. In total, 5k cycles were carried out. Before and after each stability protocol, three CV cycles between 0.05 and 1.0 V_RHE_ at a scan rate of 100 mV s^−1^ were recorded, followed by oxygen bubbling (15 min) and LSV, in a potential range between 0.05 and 1.0 V_RHE_, with a scan rate of 10 mV s^−1^ and a rotation speed of 1600 rpm.

## Figures and Tables

**Table 1:** Summary of Pt-NPs and Pt-NP@rGO in tunable aryl alkyl ionic liquids (TAAILs). Further information can be found in Table S1 in the Supplementary Information.

Sample Name X/Y = wt% Pt/rGO	TAAIL Used	wt% Pt ^1^ (X/-)	wt% rGO ^1^ (-/Y)	Average Particle Size ^2^ [nm]
**M4-1**	[BMIm][NTf_2_]	1	-	1
**M4_rGO-1/1**	[BMIm][NTf_2_]	1	1	2 ± 1
**MP4-1**	[Ph_2-Me_ImC_4_][NTf_2_]	1	-	5 ± 1
**MP4_rGO-1/1**	[Ph_2-Me_ImC_4_][NTf_2_]	1	1	3 ± 1
**MP5-1**	[Ph_2-Me_ImC_5_][NTf_2_]	1	-	4 ± 1
**MP5_rGO-2/1**	[Ph_2-Me_ImC_5_][NTf_2_]	2	1	2 ± 1
**MP9-1**	[Ph_2-Me_ImC_9_][NTf_2_]	1	-	4 ± 1
**MP9_rGO-1/0.5**	[Ph_2-Me_ImC_9_][NTf_2_]	1	0.5	3 ± 1
**DMP9-1**	[Ph_2,4-Me_ImC_9_][NTf_2_]	1	-	3 ± 1
**DMP9_rGO-1/1**	[Ph_2,4-Me_ImC_9_][NTf_2_]	1	1	6 ± 1
**MOP5-1**	[Ph_4-OMe_ImC_5_][NTf_2_]	1	-	2 ± 1
**MOP5_rGO-1/1**	[Ph_4-OMe_ImC_5_][NTf_2_]	1	1	5 ± 1

^1^ wt% in TAAIL dispersion. ^2^ Average particle sizes obtained from transmission electron microscopy (TEM) measurements. At least 200 particles were used for size determination. See Materials and Methods for more information.

**Table 2:** Electrochemical surface area (ECSA) and mass-specific activity (MA) of Pt-NP@rGO synthesized with and without IL.

Sample Name	IL Used	ECSA ^1^ [m^2^ g_Pt_^−1^]	MA ^1^ [mA mg_Pt_^−1^]
60% Pt/C	-	36 ± 11	45 ± 7
**MP4_rGO-1/1**	[Ph_2-Me_ImC_4_][NTf_2_]	35 ± 2	86 ± 7
**MP5_rGO-2/1**	[Ph_2-Me_ImC_4_][NTf_2_]	55 ± 2	101 ± 4
**MP9_rGO-1/0.5**	[Ph_2-Me_ImC_4_][NTf_2_]	33 ± 3	68 ± 12
**MOP5_rGO-1/1**	[Ph_4-OMe_ImC_4_][NTf_2_]	16 ± 5	61 ± 13
Pt-TMIM-rGO [34]	[C_12_ImC_1_][GO]	56.8	346
Pt/rGO (DMF) [35]	-	28.1	26
Pt/rGO (HYD) [35]	-	18.2	7
Pt/rGO [36]	-	14.0	92

^1^ Standard deviation obtained by three measurements (see Materials and Methods, Section 4.3).

**Table 3:** Mass-specific activity at 0.9 V of the sample **MP4_rGO-1/1** after activation and after 5k cycles.

Sample	MA [mA mg_Pt_^−1^]
Pt/C 60% activated	38
Pt/C 60% stability after 5k cycles	13
**MP4_rGO-1/1** activated	83
**MP4_rGO-1/1** stability after 5k cycles	45

## Data Availability

The data presented in this study are available on request from the corresponding author.

## Supplementary Materials

The following supporting information can be downloaded. S1. Pt-NP and Pt-NP@rGO character-ization; S2. Powder X-ray diffractograms (PXRDs); S3. Transmission electron microscopy (TEM) images of Pt-NP and Pt-NP@rGO samples; S4. TGA measurements of selected Pt@rGO samples; S5. Electrocatalytic investigations of Pt@rGO samples.
