# Supplementary material for: The Facile Deposition of Pt Nanoparticles on Reduced Graphite Oxide in Tunable Aryl Alkyl Ionic Liquids for ORR Catalysts
Source: Molecules. 2022 Feb 2;27(3):1018. doi: 10.3390/molecules27031018 (PMC8837963; doi:10.3390/molecules27031018)
Supplement: Supplementary file 1 [file molecules-27-01018-s001.zip › molecules-1537515-supplementary.pdf]

Supporting Information

The Facile Deposition of Pt Nanoparticles on Reduced Graphite Oxide in Tunable Aryl Alkyl Ionic Liquids for ORR Catalysts

Dennis Woitassek[1], Swantje Lerch[2], Wulv Jiang[3], Meital Shviro*[3], Stefan Roitsch[4], Thomas Strassner*[2], and Christoph Janiak*[1, §]

Address:

[1]Institut für Anorganische Chemie und Strukturchemie, Heinrich-Heine-Universität Düsseldorf, 40204 Düsseldorf, Germany,

[2]Physikalische Organische Chemie, Technische Universität Dresden, 01062 Dresden, Germany,

[3]Forschungszentrum Jülich GmbH, Institute of Energy and Climate Research, IEK-14: Electrochemical Process Engineering, 52425 Jülich, Germany,

[4]Institut für Physikalische Chemie, Universität zu Köln, 50939 Köln, Germany,

Email:

Christoph Janiak - janiak@uni-duesseldorf.de*

Meital Shviro m.shviro@fz-juelich.de*

Thomas Strassner thomas.strassner@tu-dresden.de*

** Corresponding author*

§ Fax: +49-211-81-12287; Tel: +49-211-81-12286

S1. Pt-NP and Pt-NP@rGO characterization

Table S1. Platinum nanoparticles (Pt-NPs) obtained in different tunable aryl alkyl ionic liquids (TAAILs) with the corresponding wt% Pt and wt% of reduced graphite oxide (rGO). For comparison, Pt-NPs in [BMIm][NTf_2] (M4) were synthesized.

Sample name X/Y = wt% Pt/rGO	IL/TAAIL	wt% Pt[a] (X/-)	wt% rGO[a] (-/Y)	Crystallite size[b] [nm]	Particle size[c] [nm]
**M4-1**	[$MImC_4$][NTf_2]	1	-	2 ± 1	1
**M4_rGO-1/1**	([BMIm][NTf_2])	1	1	2	2 ± 1
	with M = methyl group				
**MP4-1**	[$Ph_{2\text{-}Me}ImC_n$][NTf_2]	1	-	5	5 ± 1
**MP4_rGO-1/1**	with $Ph_{2\text{-}Me}$ = *ortho*-	1	1	3	3 ± 1
**MP5-1**	methylphenyl group	1	-	4	4 ± 1
**MP5_rGO-2/1**		2	1	2	2 ± 1
**MP9-1**		1	-	4	4 ± 1
**MP9-2**		2	-	3	2 ± 1
**MP9_rGO-1/0.5**		1	0.5	3	3 ± 1
**MP11_rGO-1/1**		1	1	5	5 ± 1
**DMP5_rGO-1/1**	[$Ph_{2,4\text{-}Me}ImC_n$][NTf_2]	1	1	4	5 ± 1
**DMP9-1**	with $Ph_{2,4\text{-}Me}$ = 2,4-	1	-	4	3 ± 1
**DMP9_rGO-1/1**	dimethylphenyl group	1	1	5	6 ± 1
**DMP11_rGO-1/1**		1	1	3	3 ± 1
**MOP5-1**	[$Ph_{4\text{-}OMe}ImC_5$][NTf_2]	1	-	3	2 ± 1
**MOP5_rGO-1/1**	with $Ph_{4\text{-}OMe}$ = *para*-	1	1	6	5 ± 1
**MOP9_rGO-1/1**	methoxyphenyl group	1	1	8	10 ± 2
**MOP11-1**		1	-	11 ± 1	10 ± 2
**MOP11_rGO-1/0.5**		1	0.5	9 ± 1[d]	3 ± 1

[a] wt% in TAAIL dispersion. [b] Crystallite size obtained from powder X-ray diffraction (PXRD) using Scherrer equation. [c] Average particle size from transmission electron microscopy (TEM) measurements. At least 200 particles were used for size determination. See Materials and Methods in the main document for more information. [d] Note that a significant amount of strain on the lattice structure of the Pt particles or anisotropic defects can also be responsible for peak broadening in the powder diffraction patterns.

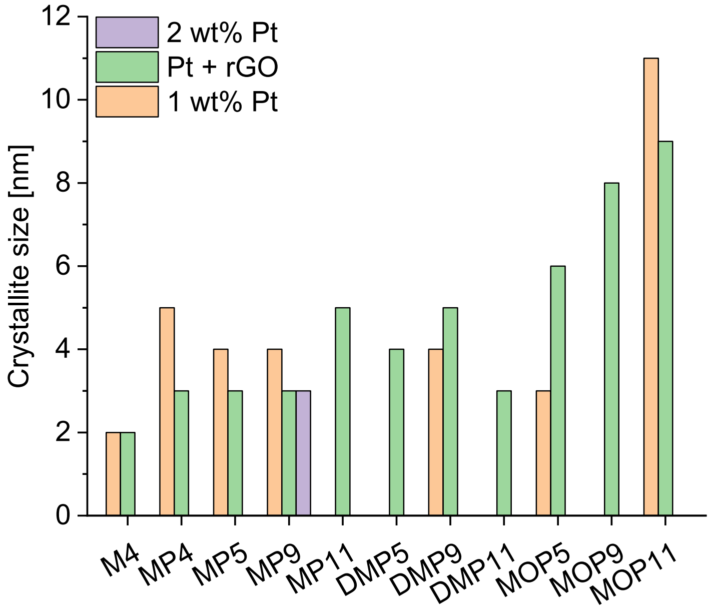

Figure S1. Crystallite sizes of Pt-NPs calculated via the Scherrer equation (Equation 1 in main text).

The synthesis and characterization of rGO can be found in the previous study of Schmitz et al. [1].

Table S2. CHNS elemental analysis of rGO and two Pt-NP@rGO samples.

	C [wt%]	H [wt%]	N [wt%]	S [wt%]	IL-content [wt%]
rGO	82.11	0.49	0.42	0.69	-
**MP4_rGO-1/1**	41.72	1.10	1.49	1.60	5
**MP5_rGO-2/1**	38.45	1.25	1.52	2.12	8
**MP9_rGO1/0.5**	39.91	1.22	1.08	1.40	6
**MOP5_rGO-1/1**	48.62	1.51	2.02	2.62	10
**MOP9_rGO-1/1**	44.05	1.18	0.75	1.11	4

The sulfur impurity in rGO can be traced back to the oxidation of graphite with $NaNO_3$, $KMnO_4$, and H_2SO_4. The increase in sulfur in Pt-NP@rGO composites is then due to the remaining traces of TAAIL with the sulfur-containing NTf_2 anion.

From the additional sulfur wt% to the carbon wt% in the composite samples, the IL content can be estimated. An IL content of 4 wt% to 9 wt% for the samples could be calculated.

Table S3. Stability of Pt-NPs after long-term storage.[a]

Sample name	TAAIL	Crystallite size [nm]	Time after synthesis
**MP4-1**	$[Ph_{2\text{-}Me}ImC_4][NTf_2]$	5	1 d
		4	1.5 a
**MP4_rGO-1/1**	$[Ph_{2\text{-}Me}ImC_4][NTf_2]$	3	1 d
		3	0.5 a
**MP5-1**	$[Ph_{2\text{-}Me}ImC_5][NTf_2]$	4	1 d
		4	1.5 a
**MP9-2**	$[Ph_{2\text{-}Me}ImC_9][NTf_2]$	3	1 d
		3	1.5 a
**MP9_rGO-1/0.5**	$[Ph_{2\text{-}Me}ImC_9][NTf_2]$	3	1 d
		4	1 a
**MP11_rGO-1/1**	$[Ph_{2\text{-}Me}ImC_{11}][NTf_2]$	5	1 d
		4	1 a
**DMP5_rGO-1/1**	$[Ph_{2,4\text{-}Me}ImC_5][NTf_2]$	4	1 d
		4	1 a
**DMP9-1**	$[Ph_{2,4\text{-}Me}ImC_9][NTf_2]$	5	1 d
		5 ± 1	0.5 a
**DMP9_rGO-1/1**	$[Ph_{2,4\text{-}Me}ImC_9][NTf_2]$	4	1 d
		4	1 a
**MOP9_rGO-1/1**	$[Ph_{4\text{-}OMe}ImC_9][NTf_2]$	8	1 d
		8	0.5 a

[a] Stored as dried material under air at room temperature.

S2. Powder X-ray diffractograms (PXRDs)

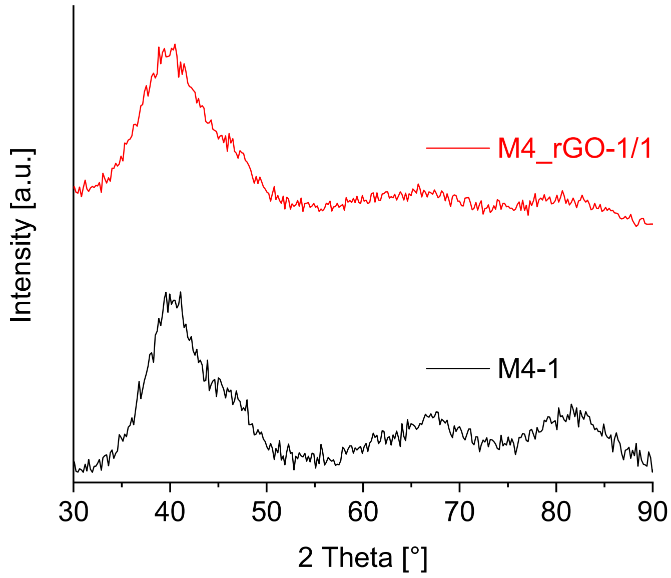

Figure S2. PXRD patterns of Pt-NPs obtained in $[BMIm][NTf_2]$ (M4) with 1 wt% Pt (black) and 1 wt% Pt/rGO (red).

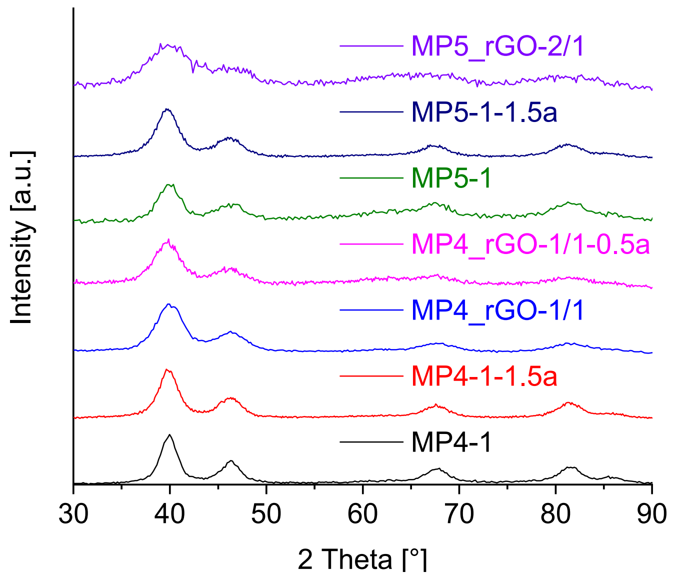

Figure S3. PXRD patterns of Pt-NPs obtained in [$Ph_{2\text{-}Me}ImC_4$][NTf_2] (MP4) with 1 wt% Pt (black), 1 wt% Pt of a 1.5-year-old sample (red), 1 wt% Pt/rGO each (blue), 1 wt% Pt/rGO each of a 0.5-year-old sample (pink), and in $Ph_{2\text{-}Me}ImC_5$][NTf_2] (MP5) with 1 wt% Pt (green), 1 wt% Pt of a 1.5-year-old sample (dark blue) and 2 wt% Pt/1 wt% rGO (violet).

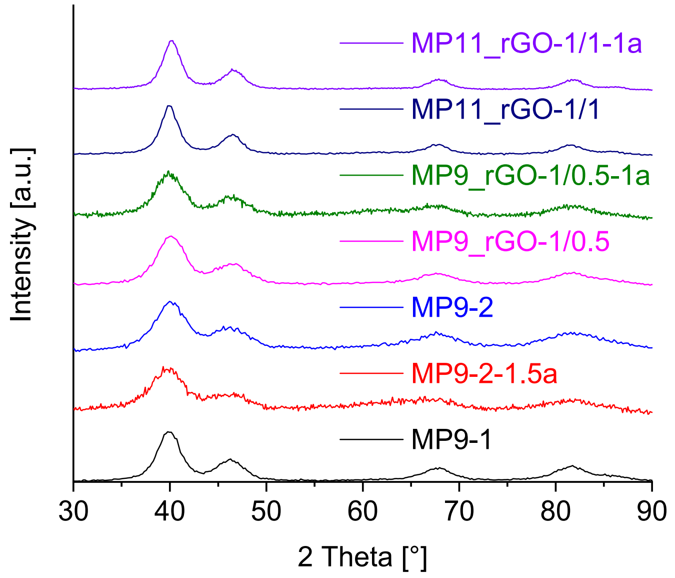

Figure S4. PXRD patterns of Pt-NP obtained in [$Ph_{2\text{-}Me}ImC_9$][NTf_2] (MP9) with 1 wt% Pt (black), 2 wt% Pt of a 1.5-year-old sample (red), 2 wt% Pt (blue), 1 wt% Pt/0.5 wt% rGO (pink), 1 wt% Pt/0.5 wt% rGO of a 1-yea- old sample (green), and in [$Ph_{2\text{-}Me}ImC_{11}$][NTf_2] (MP11) with 1 wt% Pt/rGO each (dark blue) and 1 wt% Pt/rGO each of a 1-year-old sample (violet).

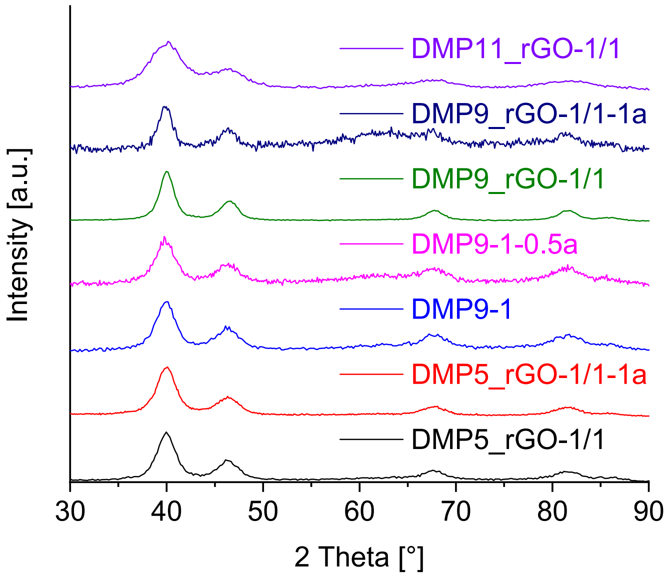

Figure S5. PXRD patterns of Pt-NP obtained in $[Ph_{2,4\text{-}Me}ImC_5][NTf_2]$ (DMP5) with 1 wt% Pt/rGO each (black), 1 wt% Pt/rGO each of a 1-year-old sample (red), in $[Ph_{2,4\text{-}Me}ImC_9][NTf_2]$ (DMP9) with 1 wt% Pt (blue), 1 wt% Pt of a 0.5-year-old sample (blue), 1 wt% Pt/rGO each (green), 1 wt% Pt/rGO each of a 1-year-old sample (dark blue), and in $[Ph_{2,4\text{-}Me}ImC_{11}][NTf_2]$ (DMP11) with 1 wt% Pt/rGO each (violet).

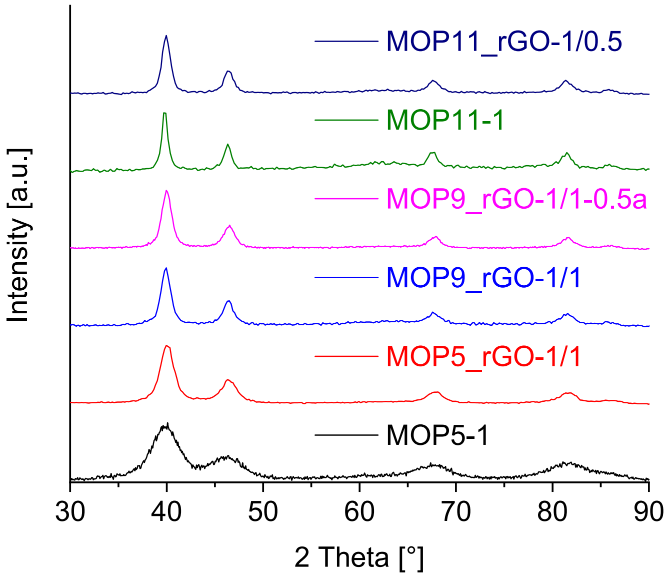

Figure S6. PXRD patterns of Pt-NP obtained in $[Ph_{4\text{-}OMe}ImC_5][NTf_2]$ (MOP5) with 1 wt% Pt (black), 1 wt% Pt/rGO (red), in $[Ph_{4\text{-}OMe}ImC_9][NTf_2]$ (MOP9) with 1 wt% Pt/rGO each (blue), 1 wt% Pt/rGO each of a 0.5-year-old sample (pink) and in $[Ph_{4\text{-}OMe}ImC_{11}][NTf_2]$ (MOP11) with 1 wt% Pt (green) and 1 wt% Pt/0.5 wt% rGO (dark blue).

S3. Transmission electron microscopy (TEM) images of Pt-NP and Pt-NP@rGO samples

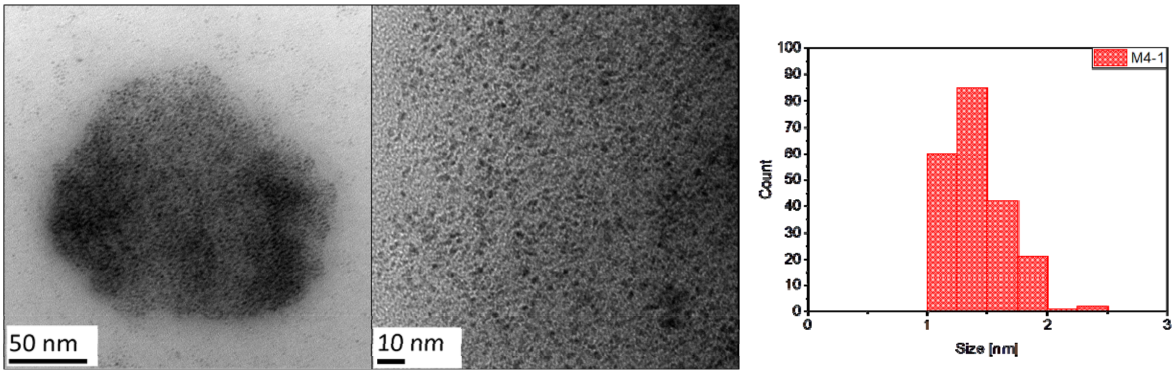

Figure S7. Sample **M4-1** obtained in [BMIm][NTf_2] (M4) with 1 wt% Pt (images with Zeiss LEO912).

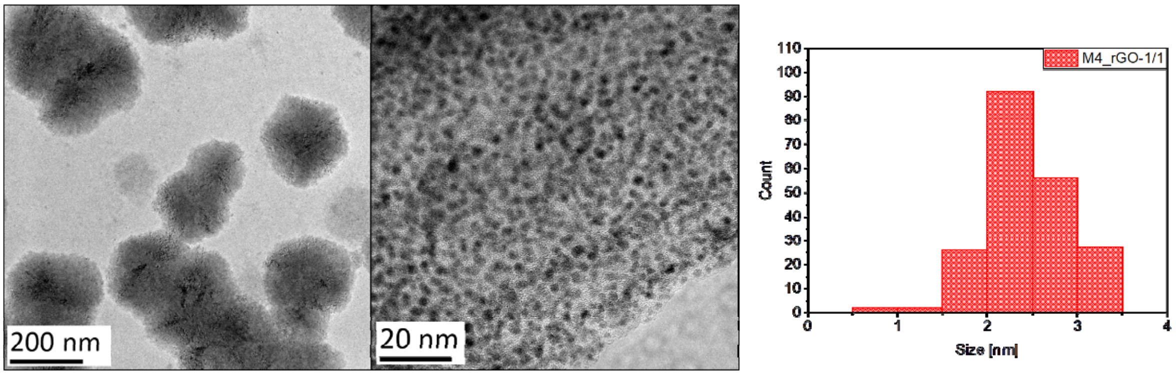

Figure S8. Sample **M4_rGO-1/1** obtained in [BMIm][NTf_2] (M4) with 1 wt% Pt/ rGO each (images with JEOL-2100 Plus).

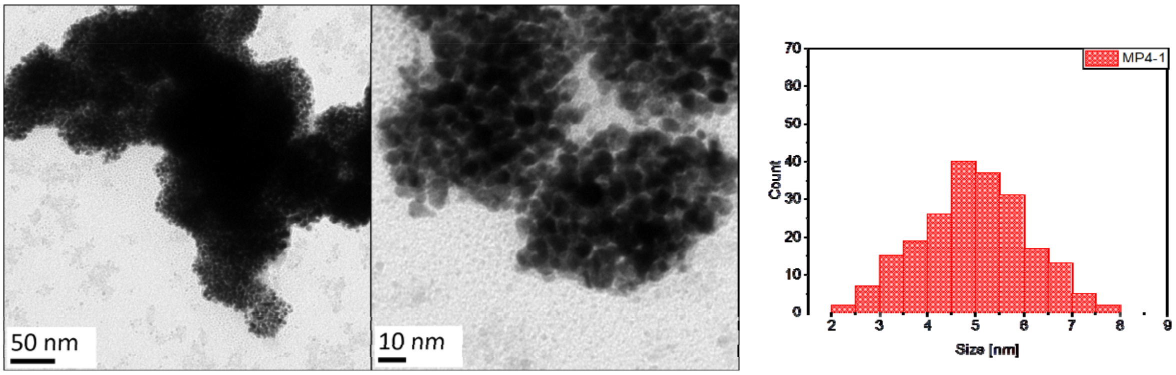

Figure S9. Sample **MP4-1** obtained in [$Ph_{2\text{-}Me}ImC_4$][NTf_2] (MP4) with 1 wt% Pt (images with Zeiss LEO912).

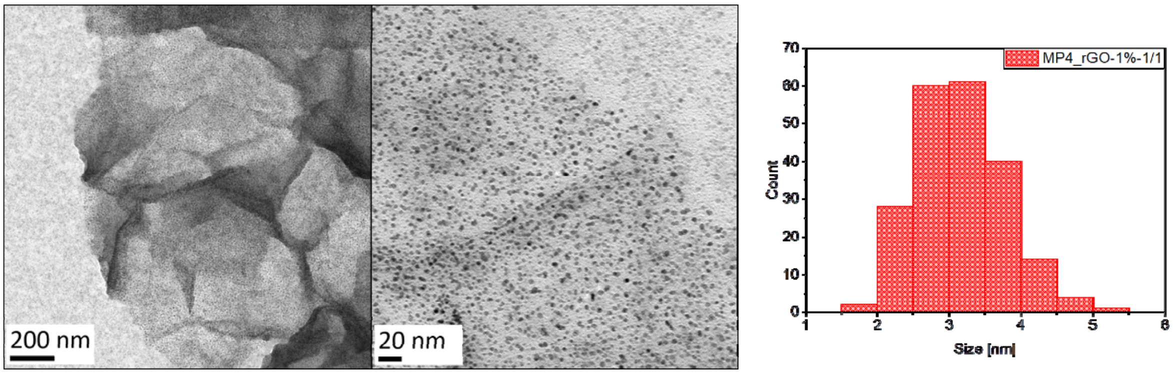

Figure S10. Sample **MP4_rGO-1/1** NP obtained in $[Ph_{2\text{-}Me}ImC_4][NTf_2]$ (MP4) with 1 wt% Pt/rGO each (images with Zeiss LEO912).

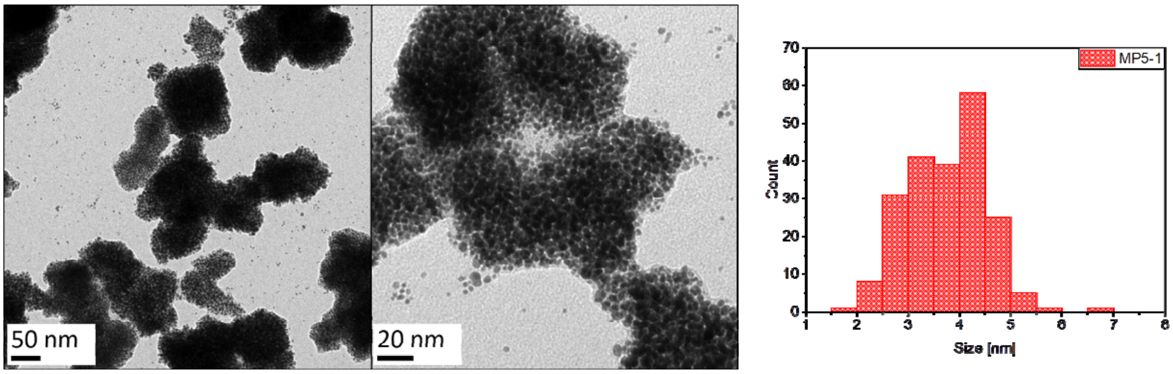

Figure S11. Sample **MP5-1** obtained in $[Ph_{2\text{-}Me}ImC_5][NTf_2]$ (MP5) with 1 wt% Pt (images with Zeiss LEO912).

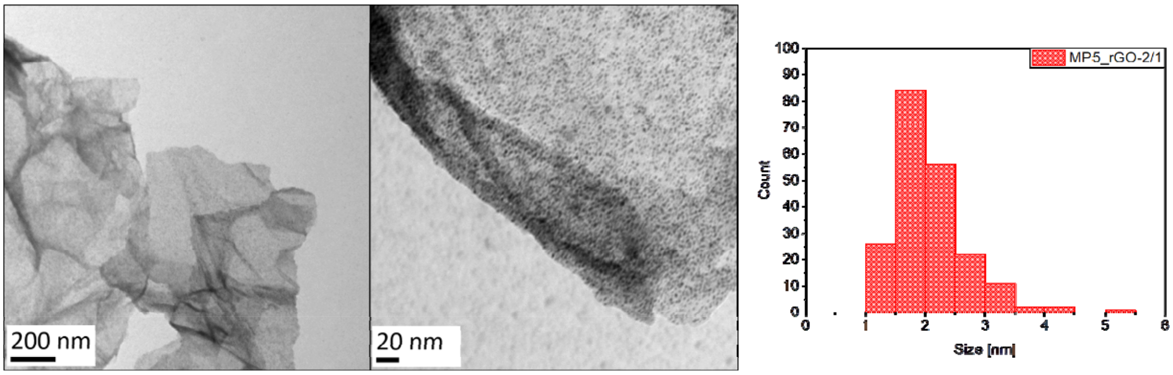

Figure S12. Sample **MP5_rGO-2/1** obtained in $[Ph_{2\text{-}Me}ImC_5][NTf_2]$ (MP5) with 2 wt% Pt/ 1 wt% rGO (images with Zeiss LEO912).

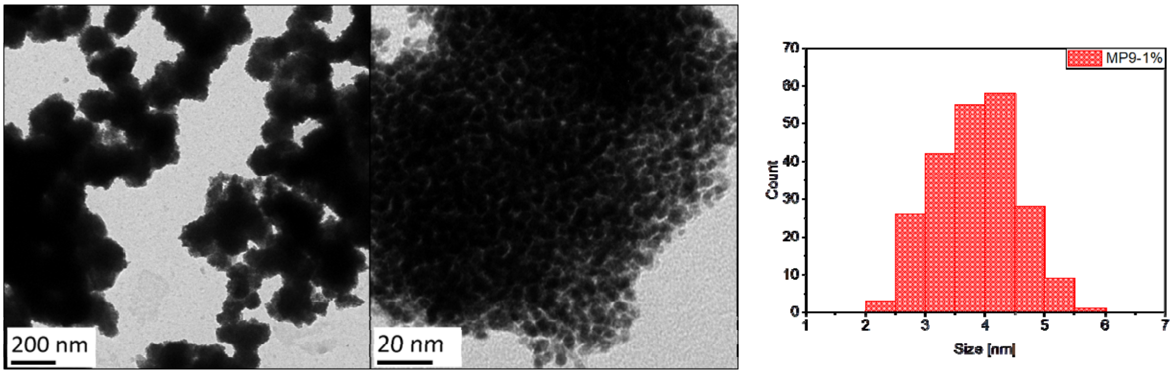

Figure S13. Sample **MP9-1** obtained in $[Ph_{2\text{-}Me}ImC_9][NTf_2]$ (MP9) with 1 wt% Pt (images with Zeiss LEO912).

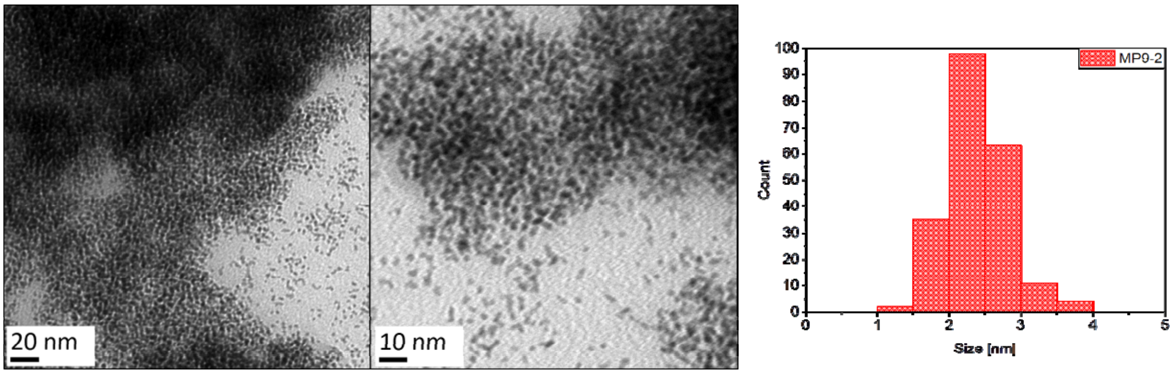

Figure S14. Sample **MP9-2** obtained in $[Ph_{2\text{-}Me}ImC_9][NTf_2]$ (MP9) with 2 wt% Pt (images with Zeiss LEO912).

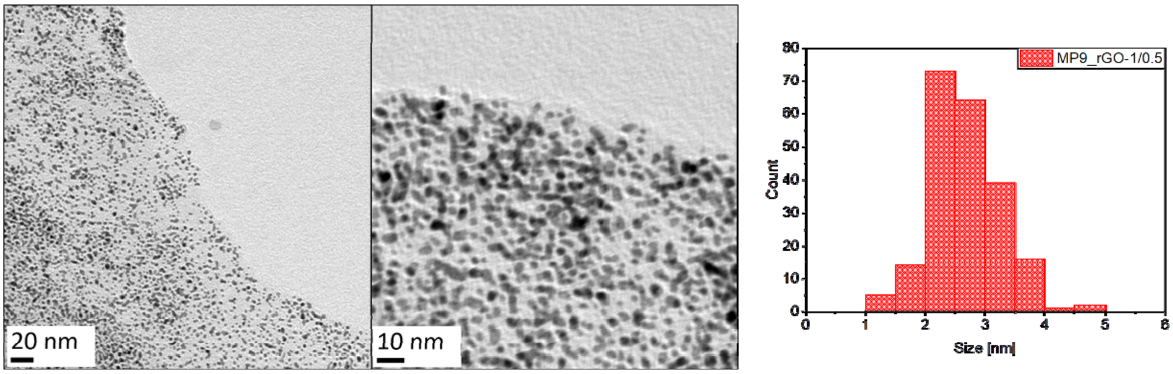

Figure S15. Sample **MP9_rGO-1/0.5** obtained in $[Ph_{2\text{-}Me}ImC_9][NTf_2]$ (MP9) with 1 wt% Pt/ 0.5 wt% rGO (images with Zeiss LEO912).

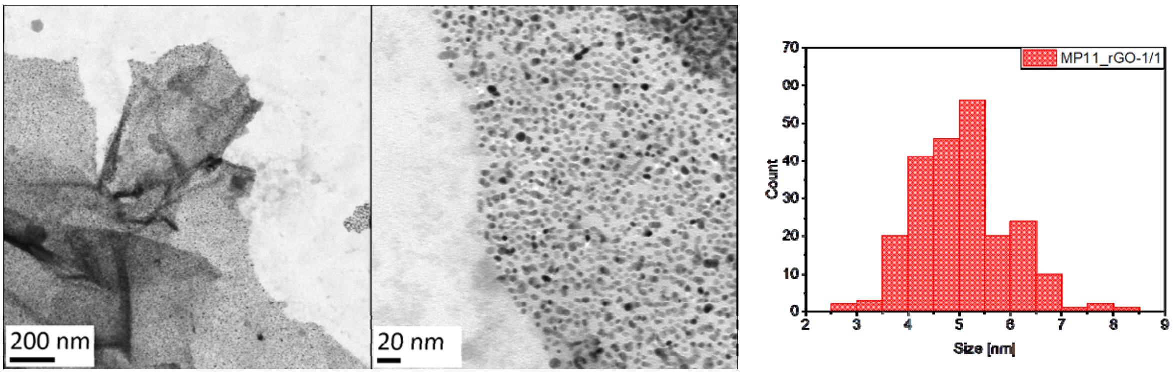

Figure S16. Sample **MP11_rGO-1/1** obtained in $[Ph_{2\text{-}Me}ImC_{11}][NTf_2]$ (MP11) with 1 wt% Pt/ rGO each (images with Zeiss LEO912).

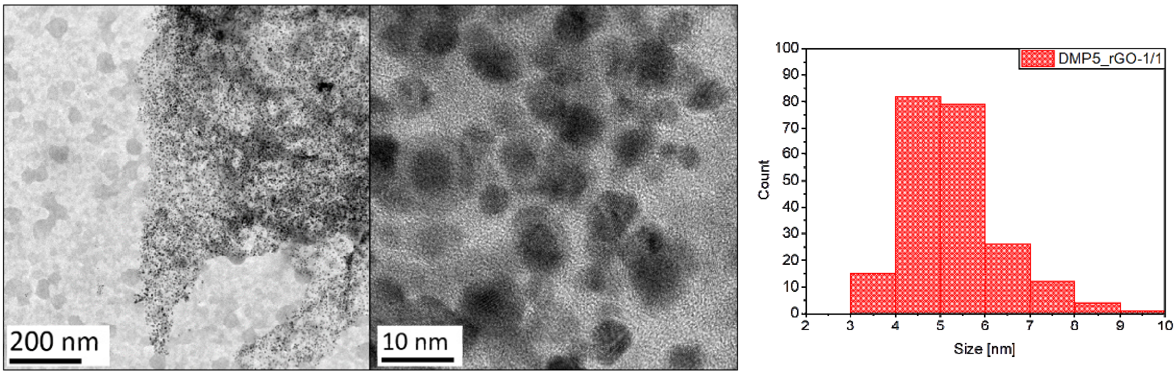

Figure S17. Sample **DMP5_rGO-1/1** obtained in $[Ph_{2,4\text{-}Me}ImC_5][NTf_2]$ (DMP5) with 1 wt% Pt/ rGO each (images with JEOL-2100 Plus).

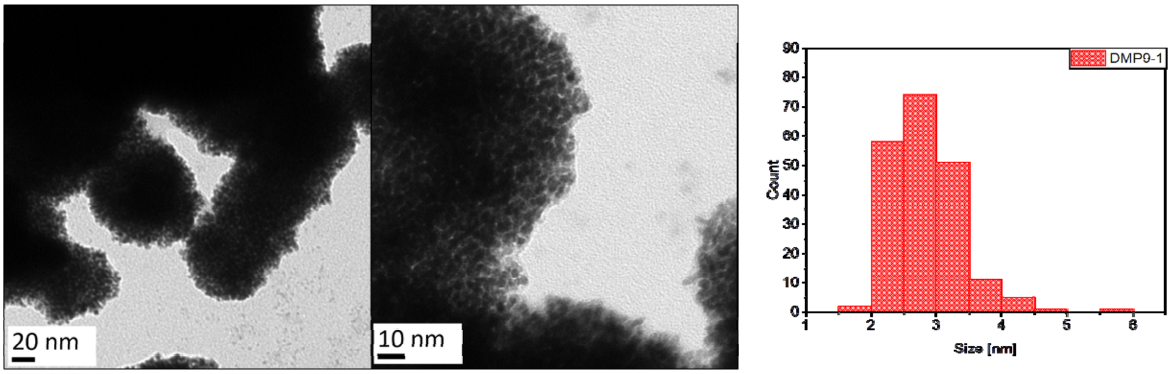

Figure S18. Sample **DMP9-1** obtained in [Ph$_{2,4\text{-Me}}$ImC$_9$][NTf$_2$] (DMP9) with 1 wt% Pt (images with Zeiss LEO912).

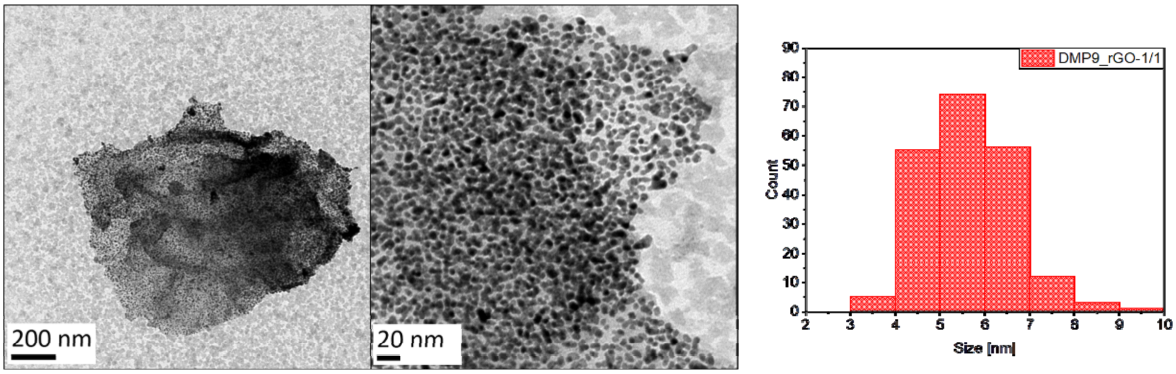

Figure S19. Sample **DMP9_rGO-1/1** obtained in [Ph$_{2,4\text{-Me}}$ImC$_9$][NTf$_2$] (DMP9) with 1 wt% Pt/ rGO each (images with Zeiss LEO912).

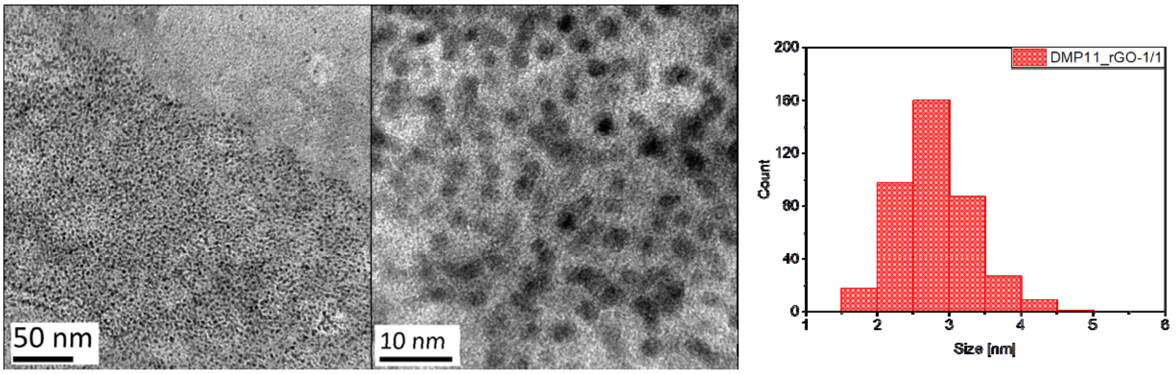

Figure S20. Sample **DMP11_rGO-1/1** obtained in [Ph$_{2,4\text{-Me}}$ImC$_{11}$][NTf$_2$] (DMP11) with 1 wt% Pt/ rGO each (images with JEOL-2100 Plus).

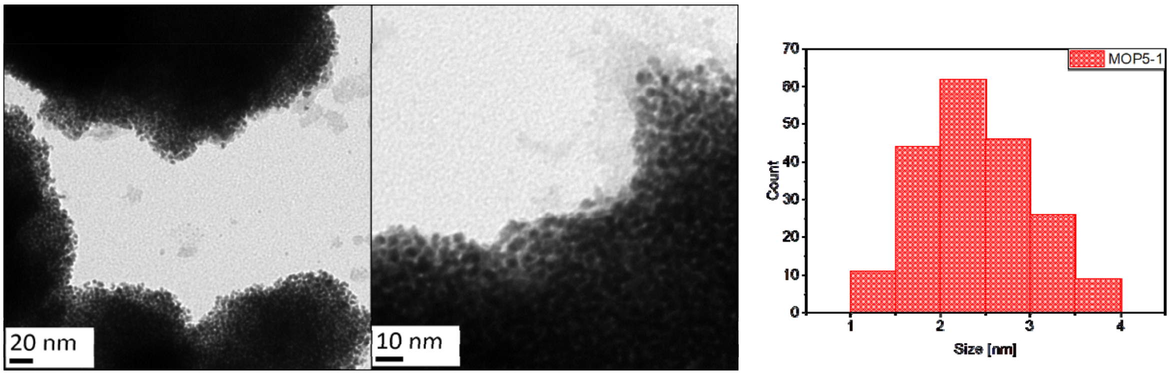

Figure S21. Sample **MOP5-1** obtained in [Ph$_{4\text{-OMe}}$ImC$_5$][NTf$_2$] (MOP5) with 1 wt% Pt (images with Zeiss LEO912).

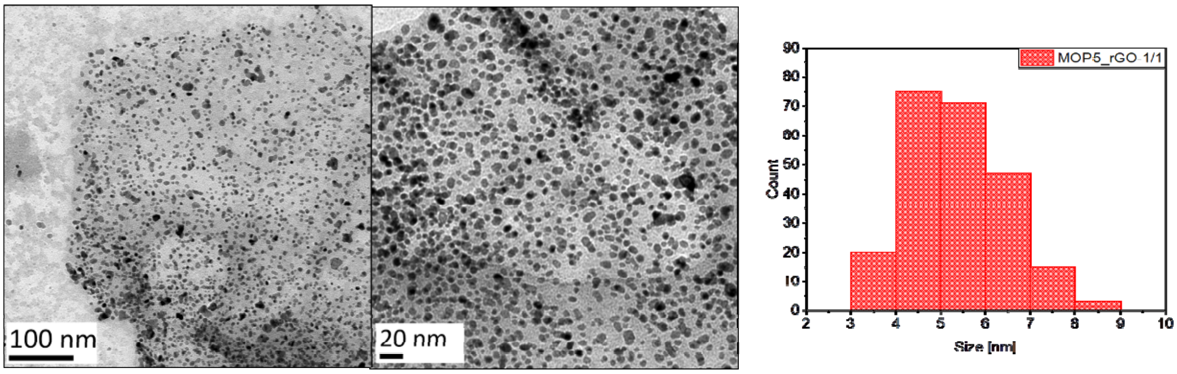

Figure S22. Sample **MOP5_rGO-1/1** obtained in [$Ph_{4\text{-}OMe}ImC_5$][NTf_2] (MOP5) with 1 wt% Pt/ rGO each (images with Zeiss LEO912).

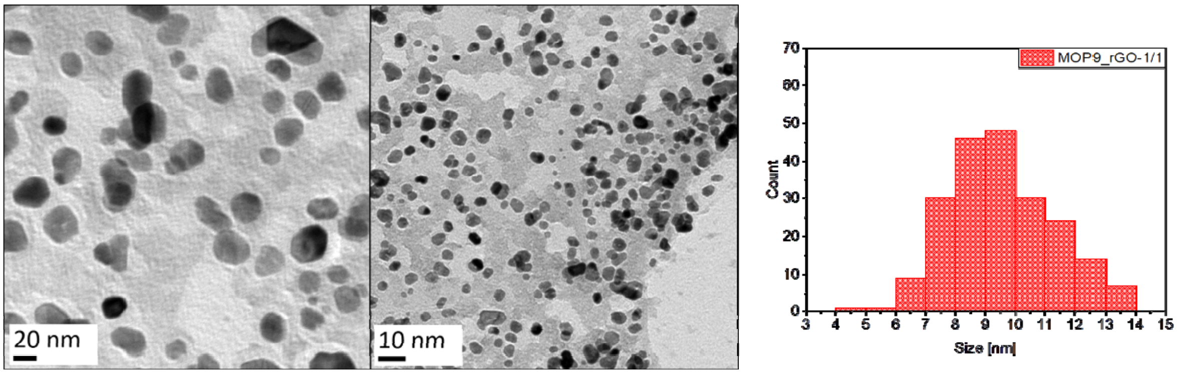

Figure S23. Sample **MOP9_rGO-1/1** obtained in [$Ph_{4\text{-}OMe}ImC_9$][NTf_2] (MOP9) with 1 wt% Pt/ rGO each (images with Zeiss LEO912).

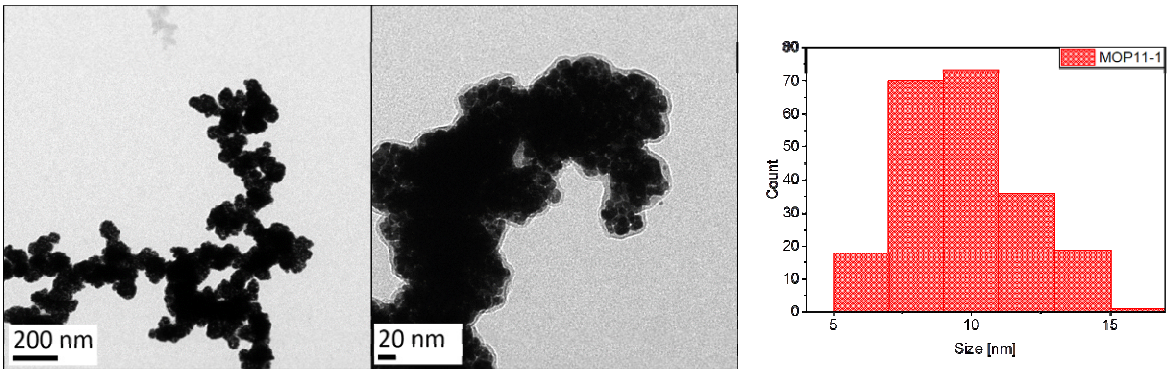

Figure S24. Sample **MOP11-1** obtained in [$Ph_{4\text{-}OMe}ImC_{11}$][$NTf_2$] (MOP11) with 1 wt% Pt (images with Zeiss LEO912).

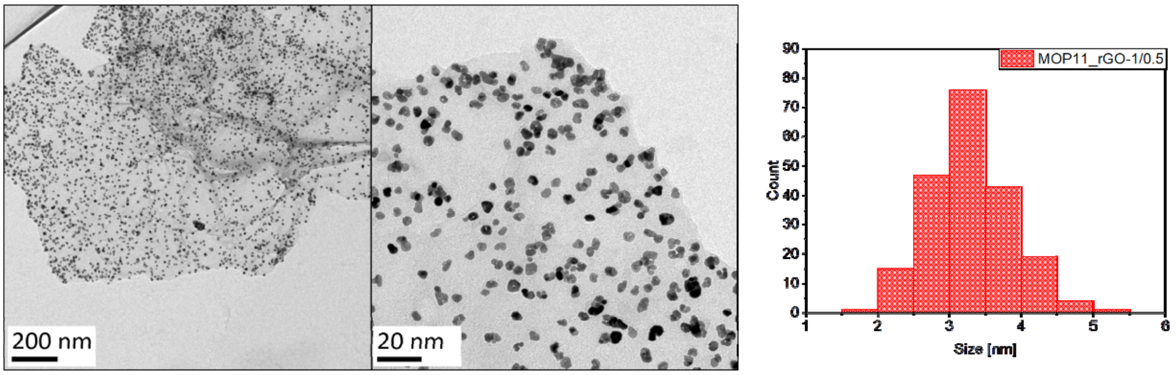

Figure S25. Sample **MOP11_rGO-1/0.5** obtained in [$Ph_{4\text{-}OMe}ImC_{11}$][$NTf_2$] (MOP11) with 1 wt% Pt/ 0.5 wt% rGO (images with Zeiss LEO912).

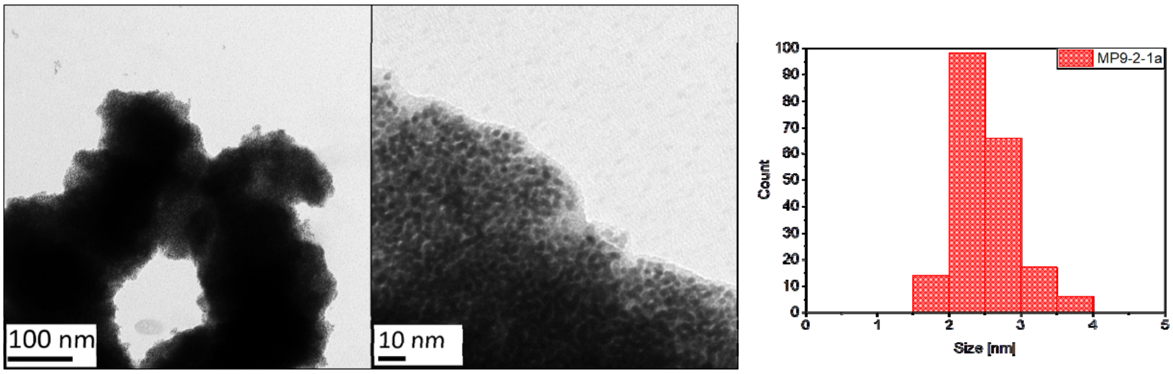

Figure S26. A 1.5-year-old sample **MP9-2** obtained in $[Ph_{2\text{-}Me}ImC_9][NTf_2]$ (MP9) with 2 wt% Pt (images with Zeiss LEO912).

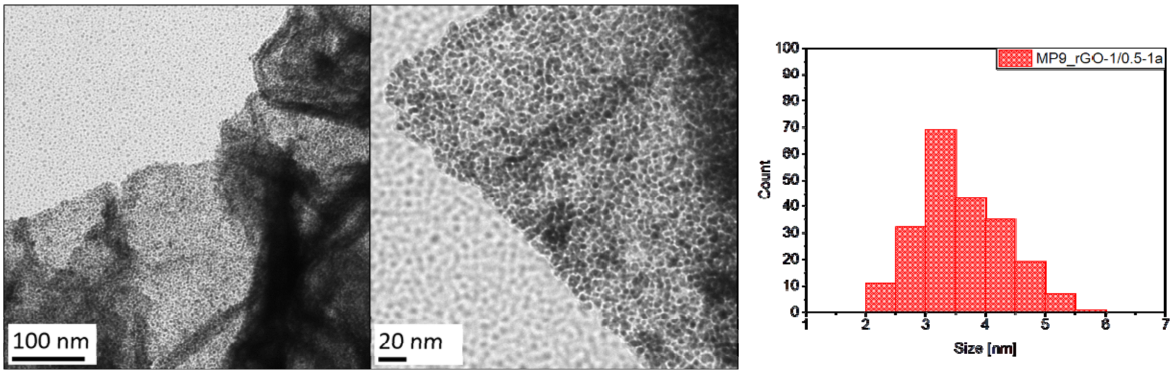

Figure S27. A 1-year-old sample **MP9_rGO-1/0.5** obtained in $[Ph_{2\text{-}Me}ImC_9][NTf_2]$ (MP9) with 1 wt% Pt/ 0.5 wt% rGO (images with Zeiss LEO912).

S4. TGA measurements of selected Pt@rGO samples

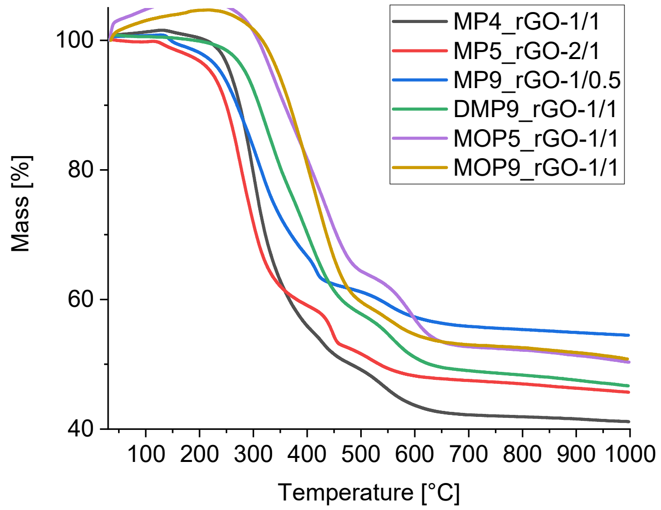

Figure S28. Thermogravimetric analysis (TGA) of the samples **MP4_rGO-1/1** (residual mass, RM = 41 wt%), **MP5_rGO-2/1** (RM = 45 wt%), **MP9_rGO-1/0.5** (RM = 54 wt%), **DMP9_rGO-1/1** (RM = 47 wt%), **MOP5_rGO-1/1** (RM = 50 wt%) and **MOP9_rGO-1/1** (RM = 51 wt%) from 30 °C to 1000 °C in synthetic air. An artifact of the TGA device, occurring when the mass of the measured sample is low, caused the increase of mass between 30 °C to 300 °C and will diminish at higher temperatures. This could not be prevented in cases where only a small amount of sample was available for the measurement.

Table S4. Estimate of rGO content.

Sample	residual mass, RM = Pt wt% [a]	100 – residual mass, wt% [b]	100 – residual mass – IL wt% = rGO estimate, wt% [c]
**MP4_rGO-1/1**	41	59	54
**MP5_rGO-2/1**	45	55	47
**MP9_rGO-1/0.5**	54	46	40
**DMP9_rGO-1/1**	47	53	44-49 [d]
**MOP5_rGO-1/1**	50	50	40
**MOP9_rGO-1/1**	51	49	45

[a] From TGA in Figure S28. [b] The difference to the residual mass (100 minus residual mass) is the sum of rGO and remaining IL in the sample. [c] From the CHNS analysis in Table S2, an IL amount of each sample was estimated. This IL wt% value was deducted from the value in the neighboring left column to estimate the wt% of rGO. [d] For this sample, no CHNS analysis was available; hence, the IL content was estimated between 4 and 9 percent, as was the range for most of the samples (cf. Table S2).

S5. Electrocatalytic investigations of Pt@rGO samples

General practice: Linear sweep voltammetry (LSV) with saturated O_2 atmosphere is sufficient to study the activity of the catalytic samples, while cyclovoltammetry (CV) with saturated O_2 atmosphere can be used to access the stability of the catalyst [2] but is not mandatory and was not used to determine the mass activity or the electrochemically active surface area of the samples mentioned in this study. See Refs. [3] and [4], which address only catalytic activity with LSV polarization curves.

The Tafel plots for all samples at 1600 rpm are included in Figure S29 in the SI, and the slopes indicate a four-electron pathway similar to the commercial platinum (Pt/C 60%). Nevertheless, the applicability of the Tafel analysis intrinsically involves questionable accuracy as most are coverage and potential-dependent. The ORR mechanism is complicated, and it is hard to calculate the number of electron transfers directly from the Tafel slope even if they are derived from the Butler–Volmer equation.

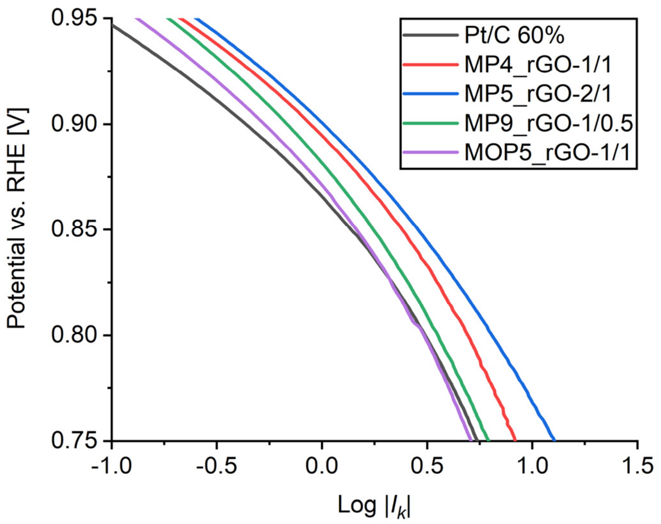

Figure S29. Corresponding Tafel plots of the Pt-NP@rGO samples derived from Figure 4b (ω = 1600 rpm).

Koutecký–Levich plots were calculated based on the following K–L equation:

$$J^{-1} = J_k^{-1} + J_d^{-1} \quad (1)$$

$$J_d = 0.62 nFD^{2/3}\omega^{1/2}v^{-1/6}c_O \quad (2)$$

where J, J_d, and J_k are the measured current density, diffusion-limited current density, and kinetic current density, respectively. ω denotes the rotation speed given by rad s^{-1}. n represents the electron transfer number, and F is the Faraday constant (96 485 C mol^{-1}). c_O is the analyte concentration (The solubility of O_2 in 0.1 M $HClO_4$ is $1.26 \cdot 10^{-6}$ mol/cm^3). D is the diffusion coefficient, for O_2 in 0.1 M $HClO_4$ it is $1.93 \cdot 10^{-5}$ cm^2/s, and v is the kinematic viscosity of the electrolyte (0.1 M $HClO_4$ $1.01 \cdot 10^{-2}$ cm^2/s. Rotation speeds of electrodes vary from 225, 400, 625, 900, 1225, and 1600 rpm. Koutecký-Levich plots are obtained based on J^{-1} vs. $\omega^{-1/2}$ at various applied potentials. The corresponding n values shown in Figure S31 below indicate a dominant four-electron pathway toward the ORR process.

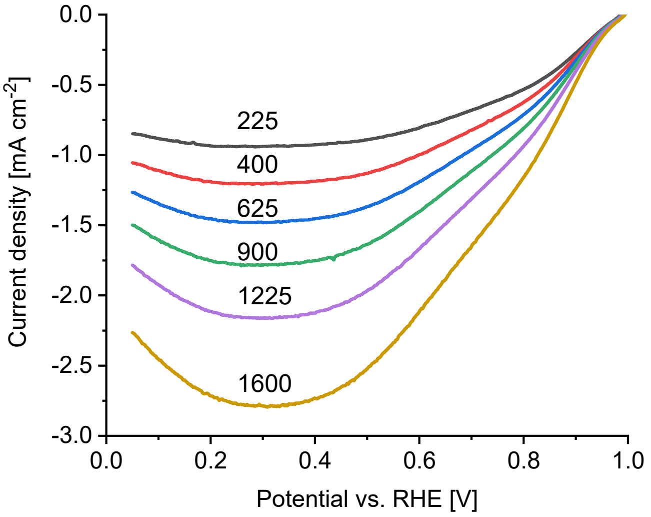

Figure S30. Oxygen reduction reaction (ORR) polarization curve (linear sweep voltammetry (LSV)) of the sample **MP4_rGO-1/1** at different rotation speeds (225 for black, 400 for red, 625 for blue, 900 for green, 1225 for violet, 1600 for yellow graph) in O_2 saturated atmosphere at 10 mV s^{-1} sweep rate.

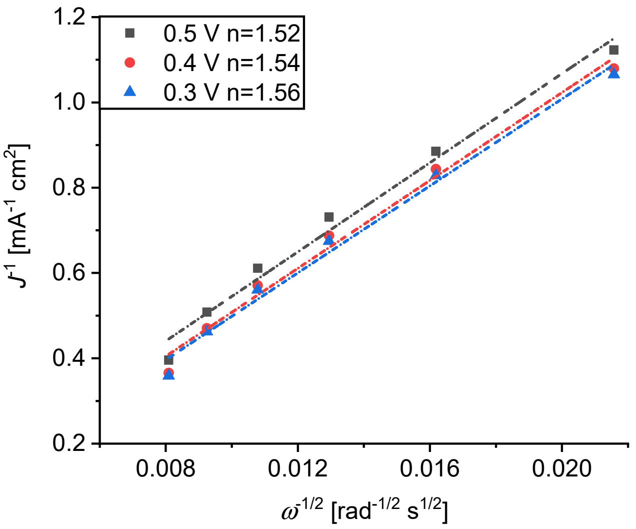

Figure S31. Koutecký–Levich plot calculated from Figure S30 at 0.3 V, 0.4 V, and 0.5 V vs. RHE. The number of transferred electrons (n) is provided in the graph legend.

References

1. Schmitz, A.; Schütte, K.; Ilievski, V.; Barthel, J.; Burk, L.; Mülhaupt, R.; Yue, J.; Smarsly, B.; Janiak, C. Synthesis of metal-fluoride nanoparticles supported on thermally reduced graphite oxide. *Beilstein J. Nanotechnol.* **2017,** *8*, 2474–2483. doi:10.3762/bjnano.8.247
2. Xia, Y.-F.; Guo, P.; Li, J.-Z.; Zhao, L.; Sui, X.-L.; Wang, Y.; Wang, Z.-B. How to appropriately assess the oxygen reduction reaction activity of platinum group metal catalysts with rotating disk electrode. *iScience* **2021,** *24*, 103024. doi:10.1016/j.isci.2021.103024
3. Sun, Y.; Polani, S.; Luo, F.; Ott, S.; Strasser, P.; Dionigi, F. Advancements in cathode catalyst and cathode layer design for proton exchange membrane fuel cells. *Nature communications* **2021,** *12*, 5984. doi:10.1038/s41467-021-25911-x
4. Wang, X.; Li, Z.; Qu, Y.; Yuan, T.; Wang, W.; Wu, Y.; Li, Y. Review of Metal Catalysts for Oxygen Reduction Reaction: From Nanoscale Engineering to Atomic Design. *Chem* **2019,** *5*, 1486–1511. doi:10.1016/j.chempr.2019.03.002
